# Bone morphogenetic protein 2-induced cellular chemotaxis drives tissue patterning during critical-sized bone defect healing: an in silico study

**DOI:** 10.1007/s10237-021-01466-0

**Published:** 2021-05-28

**Authors:** Edoardo Borgiani, Georg N. Duda, Bettina M. Willie, Sara Checa

**Affiliations:** 1 Julius Wolff Institute, Berlin Institute of Health, Charité ‐ Universitätsmedizin Berlin, Campus Virchow Klinikum, Institutsgebäude Süd/ Südstraße 2, Augustenburger Platz 1, 13353 Berlin, Germany; 2 Research Centre, Department of Pediatric Surgery, Shriners Hospital for Children-Canada, McGill University, 1003 Decarie Blvd, Montreal, QC H4A 0A9 Canada

**Keywords:** Bone defect healing, Finite element analysis, Agent-based model, Mechanobiology, Bone morphogenetic protein 2

## Abstract

Critical-sized bone defects are critical healing conditions that, if left untreated, often lead to non-unions. To reduce the risk, critical-sized bone defects are often treated with recombinant human BMP-2. Although enhanced bone tissue formation is observed when BMP-2 is administered locally to the defect, spatial and temporal distribution of callus tissue often differs from that found during regular bone healing or in defects treated differently. How this altered tissue patterning due to BMP-2 treatment is linked to mechano-biological principles at the cellular scale remains largely unknown. In this study, the mechano-biological regulation of BMP-2-treated critical-sized bone defect healing was investigated using a multiphysics multiscale in silico approach. Finite element and agent-based modeling techniques were combined to simulate healing within a critical-sized bone defect (5 mm) in a rat femur. Computer model predictions were compared to in vivo microCT data outcome of bone tissue patterning at 2, 4, and 6 weeks postoperation. In vivo, BMP-2 treatment led to complete healing through periosteal bone bridging already after 2 weeks postoperation. Computer model simulations showed that the BMP-2 specific tissue patterning can be explained by the migration of mesenchymal stromal cells to regions with a specific concentration of BMP-2 (chemotaxis). This study shows how computational modeling can help us to further understand the mechanisms behind treatment effects on compromised healing conditions as well as to optimize future treatment strategies.

**Supplementary Information:**

The online version contains supplementary material available at 10.1007/s10237-021-01466-0.

## Introduction

### Bone healing

Bone fracture healing is a regenerative process that starts autonomously when the injury occurs and it ends, if satisfactory, with a complete restoration of the original bone structure and functionality within weeks. The complexity of the healing process and the many mechanical and biological factors playing a role result in a delicate balance, which can easily result in unsuccessful healing. Many studies have investigated delayed or incomplete bone healing that might occur under compromised conditions (Augat et al. 2003; Harrison et al. 2003; Schell et al. 2008). Critical-sized defects are technically defined as those that will not heal spontaneously during the patient's lifetime (Schmitz and Hollinger 1986). Bone loss greater than 2 times the diameter of the long bone diaphysis is unlikely to result in union despite appropriate stabilization methods (Gugala et al. 2007). While it is well known that mechanical signals (i.e., strains/stresses) influence the bone healing process (Claes et al. 1997; Klein et al. 2003; Schell et al. 2005; Epari et al. 2006; Willie et al. 2011), their role in critical-sized bone defects remains largely unknown. In the early phases of healing, critical-sized bone defects are characterized by an altered mechanical loading environment within the fracture callus (Mehta et al. 2012). However, it remains so far unclear if these early tissue straining differences in critical-sized bone defects are the only reason why healing doesn’t proceed normally and why bony marrow closure occurs.

### Treatments

Treatment of critical-sized bone defects commonly consists of allo- or auto-transplantation of a bone graft inside the defect gap (Dimitriou et al. 2011). However, bone grafting is a long-term process associated with several major disadvantages. Donor site morbidity, pain, extended hospitalization time, and high risk of bleeding can result from autograft bone removal (Roberts and Rosenbaum 2012). Allograft treatments, instead, are associated with a risk of infectious diseases and a lack of bone ingrowth into the transplanted tissue, and an associated delayed osteointegration (Roberts and Rosenbaum 2012). Therapeutic strategies that involve the use of recombinant human bone morphogenetic protein 2 (rhBMP-2) have been shown to be a suitable alternative to autograft, with analog healing success rates and comparable reduced incidence of revision (Tressler et al. 2011). Besides, rhBMP-2 treatments have been reported to require shorter operative time and reduced blood loss (Tressler et al. 2011). However, excessive dosage of rhBMP-2 can lead to ectopic bone formation (Cahill et al. 2015; Krishnan et al. 2017). From a clinical point of view, the use of BMP-2 treatment is leading to inconsistent results, suggesting that its mode of action is influenced by factors related to the surgical approach. Among these factors, fixation stability might play a large role since it has been shown that mechanics strongly influence the regulation of the growth factor effectiveness (Schmidt-Bleek et al. 2016). Understanding the mechano-biological mechanisms behind BMP-2-supported bone regeneration will likely provide valuable insight into how treatment can be optimized to reduce adverse effects.

#### BMP-2

Administering exogenous BMP-2 under critical-sized bone defect conditions has been shown to promote consistent bone regeneration in preclinical models, where mechanical conditions are controlled (Schmidmaier et al. 2007; Boerckel et al. 2011; Schwarz et al. 2013, 2018). BMP-2 enhances the production of mineralized tissue inside the fracture gap (Kokubo et al. 2004; Schwarz et al. 2013) and its presence in the extracellular matrix upregulates many cellular processes including migration (Lind et al. 1996; Fiedler et al. 2002), proliferation (Knippenberg et al. 2006; Kim et al. 2013) and tissue matrix production (Knippenberg et al. 2006; Wang et al. 2011). The treatment is typically administered with an absorbable collagen sponge to provide a gradual release of the growth factor and promote bone regeneration through all the healing phases (Geiger et al. 2003; Bhakta et al. 2013; Schwarz et al. 2013; Schwarz et al. 2018). Interestingly, the tissue formation patterns after BMP-2 treatment differ from untreated bone healing (Wulsten et al. 2011; Schwarz et al. 2013; Koolen et al. 2019). While in uneventful healing progression, the bone heals by periosteal and endosteal callus formation with bone marrow opening at later stages of remodeling, a BMP-2-treated defect fully heals within few weeks, through the rapid restoration of the medullary canal (Wulsten et al. 2011; Schwarz et al. 2013; Koolen et al. 2019).

##### Mechanical stimulation

While tissue formation patterns are distinctly different in bone fracture and BMP-2-treated critical-sized defects, both processes have been reported to be mechano-sensitive; the effect of fixation stability (Epari et al. 2006; Schell et al. 2008; Röntgen et al. 2010) and injury severity (Claes et al. 1998) has been documented to influence bone healing patterns and healing time. The effect is so consistent and strong, that implants used to treat bone healing are nowadays mechanically optimized to support the healing process (Kaspar et al. 2005; Epari et al. 2006). Also in critical-sized bone defect healing, mechanical stimulation can influence the effectivity of rhBMP-2 treatment to further promote healing (Glatt et al. 2012; Schwarz et al. 2013). Mechanical stimulation enhances BMP-2 production of periosteum progenitor cells (Moore et al. 2014). From in vitro work, it is known that BMP-2 expression is stimulated by mechanical signals (Sato et al. 1999; Rauch et al. 2000) and that BMP signaling cascades are triggered by mechanical tissue straining (Kopf et al. 2012).

However, the interactions between mechanical signals, cellular responses, and bone tissue patterning during BMP-2-stimulated critical-sized bone defect healing remain still largely unknown. In most of the cases, the experimental observations of the healing outcomes are limited to the biological processes happening in vivo at the tissue level (Schwarz et al. 2013). Since it is virtually impossible to measure tissue straining in vivo in the complexity of a healing bone defect, in silico approaches may allow estimating the mechanical conditions active in such situations.

### Computer modeling

The multiscale computer approach allows to estimate the mechanical conditions within the healing region and to investigate their role on the biological processes taking place across the different scales (from subcellular to cellular to tissue level) (Bailón-Plaza and van der Meulen 2003; Isaksson et al. 2008; Geris et al. 2010; Checa et al. 2011). To our knowledge, so far only two in silico studies have investigated the mechano-biology of critical-sized bone defect healing and how it is altered under BMP-2 treatment (Moore et al. 2014; Ribeiro et al. 2015). However, none of these models have been able to predict the specific tissue formation patterns observed under BMP-2 treatments. While it is clear from experimental data that periosteal, endosteal, and intercortical bone tissue formation is observed after healing under BMP-2 conditions (Boerckel et al. 2011; Schwarz et al. 2013; Koolen et al. 2019), computer model predictions by Ribeiro et al. (2015) were limited to intercortical bone formation (Ribeiro et al. 2015). Interestingly, BMP-2 supported bone defect healing has been shown to initiate through bone defect bridging along the periosteal membrane and apparently to proceed mainly through intramembranous bone formation (Wulsten et al. 2011; Schwarz et al. 2013).

### Aim

In this study, computer models were used to investigate the mechanisms behind bone defect healing to unveil interactions at different time- and length-scales. In detail, this study aims to investigate the mechano-biological principles behind tissue formation patterns found during BMP-2-treated critical-sized bone defect healing.

## Materials and methods

### Setup

To investigate the role of BMP-2 and mechanical stimulation on critical-sized bone defect healing, we reproduced, in silico, an earlier in vivo pre-clinical model in which rhBMP-2 treatment was proven to be mechano-sensitive (Schwarz et al. 2013). In this pre-clinical study, a 5-mm-bone defect was created in the femur of rats and was monitored after: (1) local application of 50 µL (1 mg/mL) of BMP-2 on a bovine collagen sponge (Lyostypt, B. Braun), (2) external in vivo mechanical loading (500-µm displacement, rate of 10 µm/s) or the combination of the two (Schwarz et al. 2013). Four different case studies were investigated: (a) control case (no external mechanical stimulation, no exogenous BMP-2), (b) only load (external in vivo mechanical stimulation, no exogenous BMP-2), (c) only BMP-2 (no external in vivo mechanical stimulation, exogenous BMP-2), (d) BMP-2 + load (exogenous BMP-2 and external in vivo mechanical stimulation). In case studies (b) and (d), the periodical application of a 500-µm displacement of the bone in the axial direction (compression) every 7 days was simulated to replicate the external in vivo mechanical stimulation every week (Schwarz et al. 2013). For case studies (c) and (d), the initial exogenous BMP-2 dose of 50 µg in a collagen sponge support was simulated inside the osteotomy gap, reproducing the aforementioned experiment (Schwarz et al. 2013). The collagen sponge was simulated to be free of cells to reproduce the therapeutic conditions of the experimental study, where only BMP-2 was provided (Schwarz et al. 2013).

### Computer models

To unravel the role of mechanics on BMP-2 tissue patterning, we developed a multiphysics multiscale mechano-biological computer model to simulate bone healing in a critical-sized defect. A combination of finite element (FE) and agent-based computer modeling was used to investigate the interactions between the mechanical and the biological environments within the healing region (Checa et al. 2011). Two-way interaction between the models allows to iteratively determine changes in the mechanical conditions within the defect gap due to the formation of new tissues during the regeneration process. The agent-based model is cell-centered, where the behavior of each individual cell (microscale) is simulated. The distribution of cells within the bone defect regulates the tissue formation patterns (macroscale), which are iteratively updated in the FE model as changes in tissue material properties (Borgiani et al. 2015). Vice versa, the mechanical environment regulates the cellular activity. For direct comparison with microCT (µCT) data, tissue patterns at 2, 4, and 6 weeks postoperation were chosen as computer model output.

### FE model

**Fig. 1:**
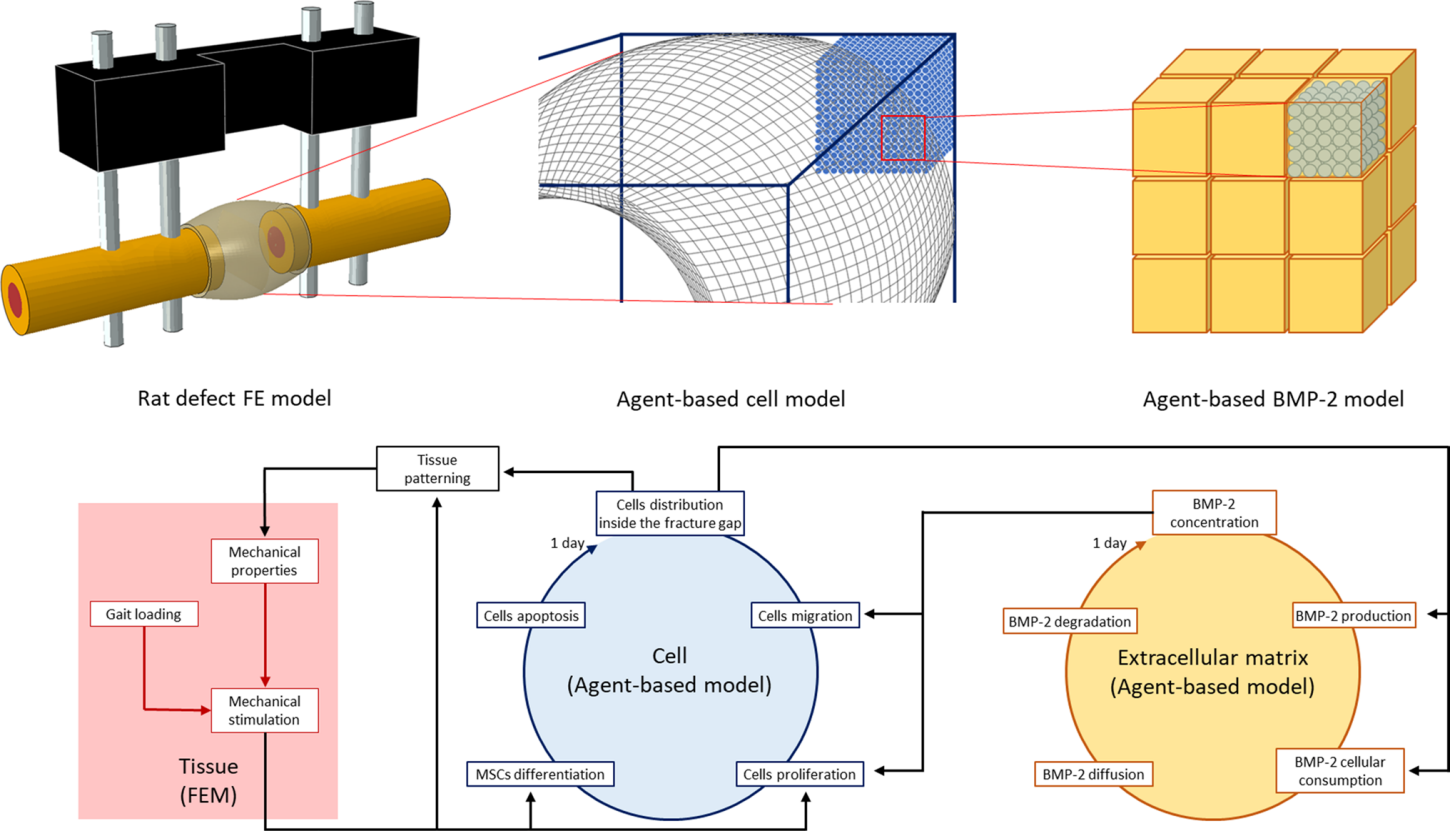
Overview of the different models used to simulate BMP-2-stimulated bone defect healing in multiple scales. Top: graphical representation of the different in silico 3D models. Bottom: flowchart of the relationship between the different models

A 3D finite element model was developed to determine the mechanical environment inside the bone defect. The FE models were created in Abaqus 6.12–2 (Simulia, Dassault Systemes) where a design tool allowed to generate the three-dimensional geometry. The model included the bone, the marrow cavity, the bone defect region surrounded by a callus growth region and the external fixator. The geometry of the femoral bone was idealized as a hollow cylinder whose internal volume represents the medullary cavity. A 5-mm-wide gap was transversally opened in the middle of the bone to reproduce the osteotomy. The callus growth region was modeled around the defect gap with dimensions obtained from in vivo data (Schwarz et al. 2013). Within this region, cartilage and bone tissue formation will simulate the growth and development of soft and hard callus, respectively. Loads and boundary conditions were applied to simulate gait in rats (Wehner et al. 2010). The FE model was mechanically loaded in the proximal bone end and constrained in the distal bone end to allow the distribution of loads along the bone shaft. A rigid external fixator was included in the FE model to stabilize the fracture and reproduce the experimental setting (Schwarz et al. 2013) (Fig. 1). The crossbar of the external fixator is temporarily removed from the model in the groups with additional mechanical stimulation (Schwarz et al. 2013).

Material properties of the different tissues are assigned to the different parts of the model (Table 1). Callus growth region material properties are initially assigned as granulation tissue. Mechanical properties of the external fixator are based on the actual materials of the fixator used experimentally (Schwarz et al. 2013).

**Table 1:** Mechanical properties of different materials assigned to the finite element model

	Callus growth region (granulation tissue)	Bone cortex	Bone marrow	Fixator structure (PEEK)	Fixator nails (Titanium)	Fibrous tissue	Cartilage	Bone
Young’s modulus (MPa)	0.2	5000	2	3800	110,000	2	10	5000
Poisson’s ratio (−)	0.167	0.3	0.167	0.3	0.3	0.167	0.3	0.3
Permeability (m^4^/N s * 10^–14^)	1	0.001	1	–	–	1	0.5	37
Bulk modulus grain (MPa)	2300	13,920	2300	–	–	2300	3700	13,940
Bulk modulus fluid (MPa)	2300	3200	2300	–	–	2300	2300	2300

Adapted from Checa et al. (2011). PEEK = PolyEtherEtherKetone

The meshing was performed with three-dimensional eight-node brick poroelastic elements (C3D8P) with an average size of 0.50 mm (0.25 mm inside the callus growth region, the region of investigative interest).

### Agent-based cellular model

To investigate the role of cellular activity on tissue patterning, a 3D agent-based computer model was developed. Each agent represented a cell, and a matrix of possible cell positions was created to investigate the spatial distribution of cells inside the osteotomy gap (Fig. 1). Four different cellular phenotypes are considered: mesenchymal stromal cells (MSC), fibroblasts, chondrocytes, and osteoblasts. To initialize the agent-based model, MSCs were randomly seeded in the medullary cavity and around the cortical periosteum. During the initialization phase, 30% of the cell positions available in the marrow and periosteum regions were assigned to MSCs (Checa et al. 2011). Inside the callus growth region, cells migrate, proliferate, differentiate, and experience apoptosis (Checa et al. 2011). The cellular activity ratios are reported in Table 2.

**Table 2:** Cellular activity ratios

Cell phenotype	Migration rate (μm/h)	Proliferation rate (day^−1^)	Differentiation rate (day^−1^)	Apoptosis rate (day^−1^)
MSC	30	0.60	0.30	0.05
Fibroblast	30	0.55	–	0.05
Chondrocyte	–	0.20	–	0.10
Osteoblast	–	0.30	–	0.16

Adapted from Checa et al. (2011)

The MSC differentiation is regulated by the mechanical environment inside the osteotomy gap predicted by the FE model (Prendergast et al. 1997). Moreover, the mechanical environment determines the regions where the cells can proliferate and are subject to apoptosis according to their phenotype.

Furthermore, in line with experimental observations showing bone healing arrest after few days in critical-sized bone defects when left untreated (Chaubey et al. 2013; Schwarz et al. 2013; Skaliczki et al. 2013), we investigated the role of limited cellular recruitment on the bone healing outcome. We simulated two different cases:

1. Continuous cellular recruitment through the whole healing period: MSC migration and proliferation are allowed during the whole healing process.
2. Limited cellular recruitment: MSC migration and proliferation ratios were set to zero after 10 days. Differentiation and apoptosis ratios were kept unaltered.

### FE mechanical environment regulates cell differentiation

To simulate the dynamical progression of bone defect healing at cellular level, undifferentiated MSCs adapted their phenotypes according to the surrounding mechanical stimulation. The mechanical environment determined using the FE model regulated the spatial differentiation of MSCs. The differentiation rule proposed by Prendergast et al. (1997) was implemented, who described the differentiation stimulus ($S$) as a linear combination of octahedral shear strain ($\gamma$) and fluid flow velocity ($\upsilon$):

1 $$S = \frac{\gamma }{0.0375} + \frac{\upsilon }{3{ \mu}\; {\text{m\,s}}^{ - 1}}$$

While fluid flow velocity is directly obtained from the FE model, the octahedral shear strain is a combination of minimal principal strains (${e}_{xx}$) and shear strains (${e}_{xy}$):

2 $$\gamma = \frac{2}{3} \sqrt {\left( {e_{11} - e_{22} } \right)^{2} + \left( {e_{22} - e_{33} } \right)^{2} + \left( {e_{11} - e_{33} } \right)^{2} + 6e_{12} + 6e_{23} + 6e_{13} }$$

MSCs in the agent-based model differentiate according to the stimulus $S$ according to the ranges in Table 3.

**Table 3:** MSC differentiation ranges according to the mechanical stimulus ($S$)

Mature osteoblast	Immature osteoblast	Chondrocyte	Fibroblast
$S$≤ 2.53	2.53 < $S$ ≤ 3.00	3.00 < $S$ ≤ 5.00	$S$> 5.00

Derived from Checa et al. (2011)

### Cell distribution determines tissue patterning

The distribution of cells regulates tissue patterning within the callus growth region. Each cell is able to produce a specific tissue matrix, according to its phenotype (fibroblasts—fibrous tissue, chondrocytes—cartilage, osteoblasts—bone), and the agent-based available positions around each cell are assigned to tissue elements according to cell-specific production rates (Isaksson et al. 2008) (Table 4).

**Table 4:** Tissue production and degradation ratios

Tissue	Production rate (μm^3^/cell/h)	Degradation rate (μm^3^/cell/h)
Fibrous tissue	5000	5000
Cartilage	5000	5000
Bone	3000	3000

Adapted from Isaksson et al. (2008)

Similarly, tissue elements are subject to removal according to specific matrix resorption rates (Isaksson et al. 2008) (Table 4). At every iteration, the agent-based model is updated to simulate both cellular and tissue patterning dynamics within the defect. The resulting tissue patterning predicted at each iteration is used to update the material properties of the callus. At each iteration, the material properties of every element in the callus FE model are averaged according to the tissue presence within it. The material properties of each tissue are reported in Table 1.

### Agent-based model of BMP2 concentration dynamics

A second agent-based model was developed to simulate the dynamics of BMP-2 concentration inside the callus. In this model, each agent of the three-dimensional matrix is associated with the concentration of growth factor in that specific position. This BMP-2 agent-based model is characterized by larger agents than the cellular model and each agent of the BMP-2 model could contain a maximum of 125 cells (5 × 5 × 5) (Fig. 1). Double-way interaction between the two agent-based models was then implemented: the cells that occupy the volume of a BMP-2 agent regulate its BMP-2 production and consumption and, vice versa, the BMP-2 concentration of the agent regulates the activity of all the cells contained within. A system of discrete equations describes the dynamics of BMP-2 following Ribeiro et al. (2015). Production and consumption of BMP-2 are regulated by the spatial presence of MSCs and bone cells (Ribeiro et al. 2015):

3 $$\Delta \left[ {{\text{BMP}}} \right] = { }\left[ {\frac{\alpha }{{\gamma \left[ {{\text{BMP}}} \right] + { }\gamma_{0} }} - \frac{{V_{{K{ }}} \left[ {{\text{BMP}}} \right]}}{{K_{M}^{A} + \left[ {{\text{BMP}}} \right]}}} \right]*\left( {n_{{{\text{MSC}}}} + n_{{{\text{OB}}}} } \right){*}\Delta t$$

Equation 3 represents the local variation of BMP-2 concentration in a discrete period of one computer iteration ($\Delta t$= 2 h). The concentration of BMP-2 ($\left[\mathrm{BMP}\right]$) varies inside each domain of the BMP-2 agent-based model according to the number of MSCs (${n}_{MSC}$) and osteoblasts (${n}_{\mathrm{OB}}$) spatially included inside it. The positive part of the equation quantifies the cellular production of BMP-2, while the negative one reproduces its reduction due to cellular consumption. $\alpha$, $\gamma$, ${\gamma }_{0}$, ${V}_{K}$, ${K}_{M}^{A}$ are parameters that characterize the production and consumption of BMP-2 (Table 5).

**Table 5:** Parameter values used to simulate BMP-2 production and consumption dynamics

Parameter	Value
$\alpha$	2 × 10 ^−9^ ng cm^−3^ cell ^−1^ day ^−1^
$\gamma$	15 cm^3^ ng ^−1^
${\gamma }_{0}$	0.01
${V}_{K}$	1.43 × 10 ^−7^ ng cm ^−3^ day ^−1^ cell ^−1^
${K}_{M}^{A}$	11.01 ng cm ^−3^

Adapted from Ribeiro et al. (2015)

BMP-2 is also subject to degradation. An exponential decay is considered in the model with a half-life of ${t}_{1/2}$ = 0.42 day (Bramono et al. 2012):

4 $$\Delta \left[ {{\text{BMP}}} \right] = \left[ {{\text{BMP}}} \right]{\text{*exp}}\left( { - \ln \left( 2 \right)/t_{1/2} { * }\Delta t} \right)$$

The diffusion of BMP-2 follows Fick’s law; i.e., BMP-2 diffuses from high- to low-concentration regions with diffusivity *D* = 8.64 × 10 ^−2^ cm^2^/day (Ribeiro et al. 2015). Cellular production, consumption, BMP-2 degradation, and diffusion regulate the distribution of BMP-2 in space and time. At every iteration, in every agent of the BMP-2 model, the BMP-2 concentration is updated.

### BMP-2 regulated cellular activities

**Fig. 2:**
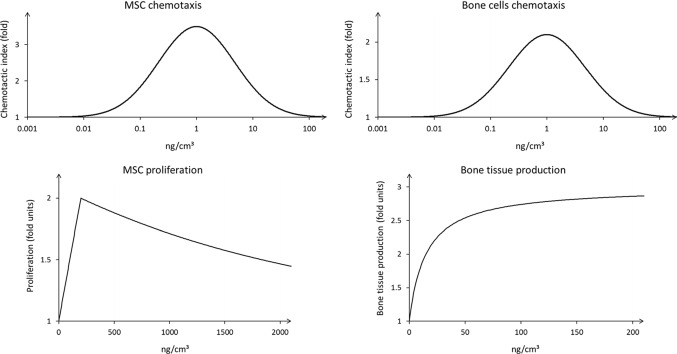
BMP-2 chemotactic effect on MSC and osteoblast migration (top), enhancement of MSC proliferation capacity (left bottom), and osteoblast bone tissue production (right bottom). Adapted from Ribeiro et al. (2015)

In our computer model, BMP-2 enhances the progression of bone healing by upregulating biological processes at cellular and tissue levels. At the cellular level, BMP-2 promotes MSC and osteoblasts chemotaxis. Based on the literature (Lind et al. 1996; Fiedler et al. 2002), chemotaxis was modeled to be maximum at a BMP-2 concentration of 1 ng/cm^3^ (Fig. 2). At this concentration, MSC and osteoblast chemotaxis indexes are 3.5 and 2.2 times higher than under no BMP-2 treatment, respectively (Fig. 2) (Fiedler et al. 2002). In the model, the chemotaxis index regulates the random migration of the cells, providing a preferential movement toward the direction where the index is higher. With chemotactic regulation, migration happens according to a weighted randomization algorithm, where the weight for each direction depends on the chemotaxis index in that direction. This means that there is a higher probability that the migrating cell moves in the direction where the chemotaxis index is higher. Also, the presence of BMP-2 was modeled to promote the proliferation of MSCs with a peak of a twofold increase in proliferation rate at the concentration of 200 ng/cm^3^ (Fig. 2) (Knippenberg et al. 2006; Kim et al. 2013). At the tissue level, the effect of BMP-2 on bone formation was modeled by an upward asymptotic behavior, similar to Ribeiro et al. (2015). The BMP-2 treatment effect was modeled by a threefold increase in bone production at high BMP-2 concentrations (Wang et al. 2011) (Fig. 2).

### Collagen sponge

To simulate the presence of the collagen sponge used as a BMP-2 carrier in the in vivo pre-clinical experiment (Schwarz et al. 2013), BMP-2 within the fracture gap is gradually dosed throughout the entire simulation. The collagen sponge was simulated to gradually release BMP-2 as reported experimentally (Fujioka-Kobayashi et al. 2015). A second-grade polynomial is used to interpolate experimental values of BMP-2 retention into collagen sponges. Collagen sponge residual BMP-2 mass (${m\left(\mathrm{BMP}\right)}_{sp}\left(t\right)$) decreases from the initial mass (${m\left(\mathrm{BMP}\right)}_{sp}\left(0\right)$= 50 µg in this model) according to the following time-dependent function (t in minutes) (Fujioka-Kobayashi et al. 2015):

5 $$m\left( {{\text{BMP}}} \right)_{sp} \left( t \right) = m\left( {{\text{BMP}}} \right)_{sp} \left( 0 \right){*}\left( {68{\text{*exp}}\left( { - 0.012{*}t} \right) + 22.1{\text{*exp}}\left( { - 0.00006{*}t} \right)} \right)$$

Residual BMP-2 is not affected by cellular production, cellular consumption, or diffusion. Also, residual BMP-2 does not regulate cellular processes. Residual BMP-2 is subject to slower degradation and its dynamics follows an exponential decay with a longer half-life (${t}_{1/2}$ = 3.25 day) (Ribeiro et al. 2015) than free BMP-2. Only free BMP-2 plays an active role in the bone healing model. A cylindrical region was simulated to describe the sponge domain, which shape filled the bone defect gap.

To investigate the role of BMP-2 slow release from the collagen sponge, an additional model was created where the whole amount of BMP-2 is considered to be released within the fracture gap since the first iteration: “fast release model”. The BMP-2 agent-based model is initialized with a homogenous concentration of free BMP-2 within the osteotomy gap. It is subjected over time to cellular production, cellular consumption, diffusion, and degradation and was available for the regulation of cellular processes since the first stages of healing. This case was only simulated for the BMP-2 treatment case without mechanical stimulation.

### Outcome of the simulations

For comparison with in vivo data, computer model simulation outcomes are focused on the dynamics of bone tissue patterning and the quantification of bone tissue volume throughout healing. To represent bone tissue patterning, the callus growth region is divided into small cubic voxels of 0.04 mm size (6.4 × 10^–5^ mm^3^ volume). A Boolean value is then assigned to each voxel representing “bone” or “not-bone.” The voxel is assigned to “bone” only when the model predicted that more than half of its volume was filled with bone tissue. In the in silico figures presented in this manuscript, only bone voxels are visible (in gray). The mineralized callus volume (Bone Volume, BV) is calculated as a product between the number of “bone” voxels times the voxel volume. The bone cortices are excluded from this quantification.

## Results

### Limited cellular recruitment can explain non-union in critical-sized bone defect healing

**Fig. 3:**
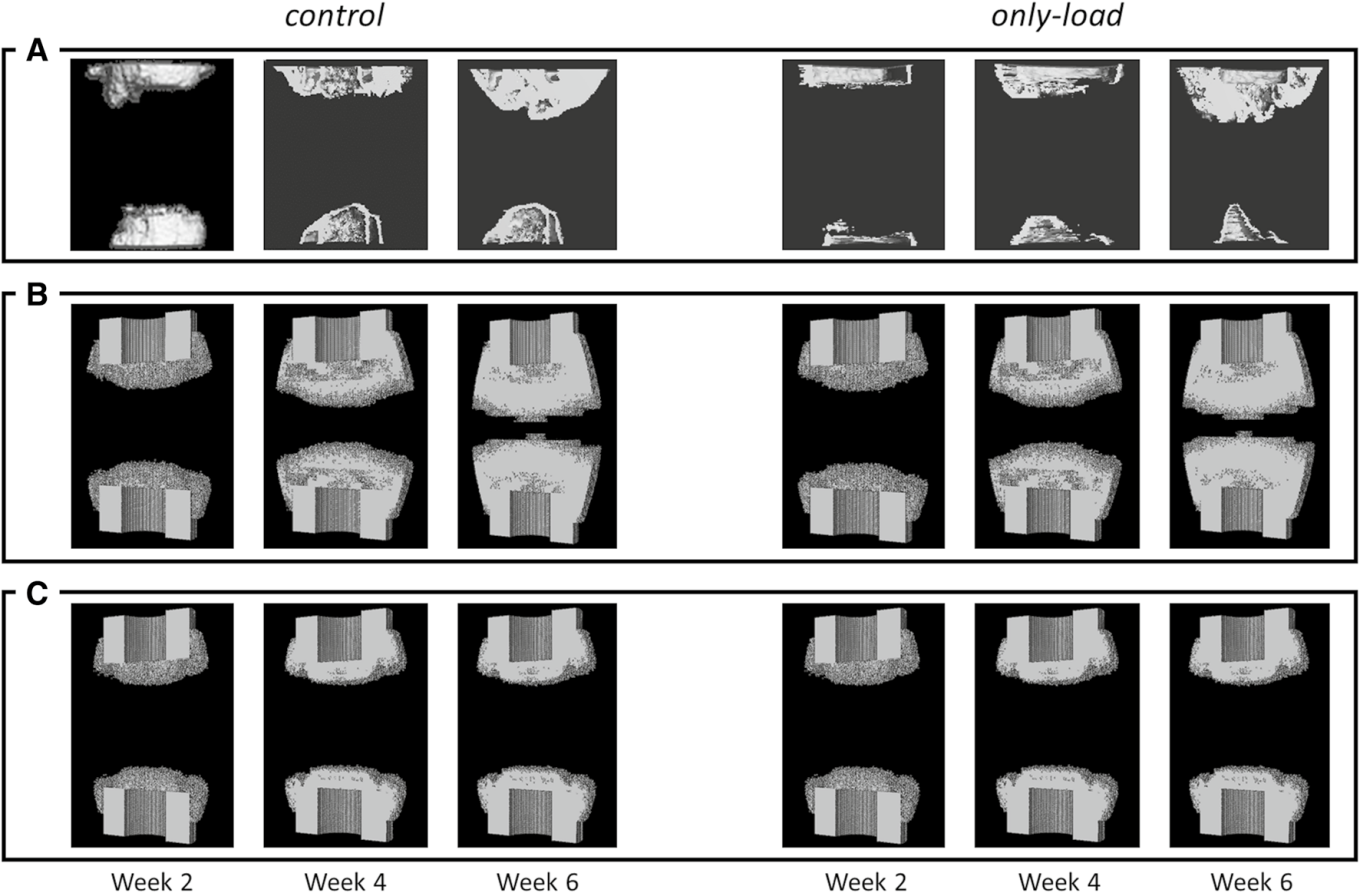
Bone tissue patterning of critical-sized defect healing under untreated conditions (control: no external mechanical stimulation, no BMP-2; and only-load: external in vivo mechanical stimulation, no BMP-2) cases at 2, 4 and 6 weeks postoperation. Comparison between in vivo µCT images (Schwarz et al. 2013) (**A**) and in silico predictions under continuous (**B**) and limited (**C**) cellular recruitment conditions

Untreated critical bone defect healing models (control and only-load) predicted incomplete healing at 6 weeks postoperation. Experimental µCT images showed encapsulation of the bone marrow cavity at 2 weeks postoperation, which was observed to consolidate at later time-points (Schwarz et al. 2013) (Fig. 3a). At 6 weeks, a non-union was observed in vivo under both untreated conditions (Schwarz et al. 2013) (Fig. 3A). If continuous recruitment of MSC during the whole healing period was modeled, in silico predictions lead to considerable endosteal and periosteal bone formation at 2 weeks (Fig. 3B). This leads to almost a closure of the defect at 6 weeks, in contrast to µCT observations (Fig. 3A). If cellular migration and proliferation were modeled as being limited to the first 10 days postoperation, computer model predictions compared well to µCT observations and predicted non-unions in non-BMP-2 treated cases (control and only-load) (Fig. 3C). Bone formed a capsule of the medullary cavity and thereafter stopped forming across the gap (Fig. 3C).

**Fig. 4:**
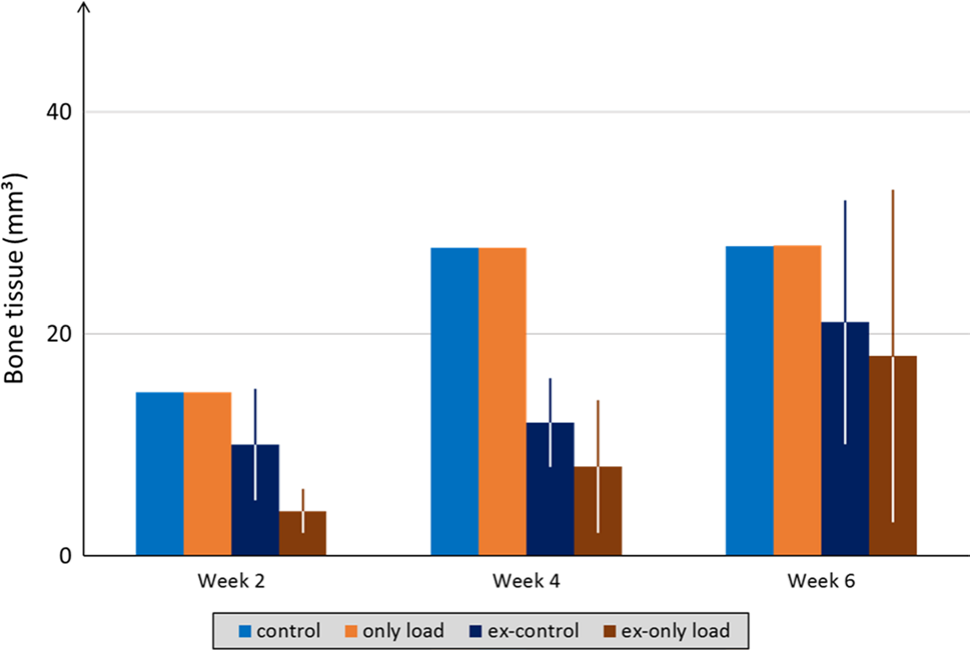
Bone tissue volume comparison between in silico (average) and in vivo (BV average ± SD) within the healing region at 2, 4, and 6 weeks postoperation for untreated case scenarios (control and only-load). Note: “ex-” prefix in legend identifies in vivo

Both in vivo and in silico results showed that without BMP-2 treatment, bone healing progression was not enhanced by weekly external loading. The tissue formation dynamics, which also led to incomplete defect regeneration, were similar to the ones observed in the control case both in µCT (Schwarz et al. 2013) (Fig. 3A) and in silico (Fig. 3C). Marrow cavity encapsulation was still predicted at 6 weeks postoperation (Fig. 3C). Experimentally, it was shown that a non-significant increase in BV was observed at all time-points when additional mechanical stimulation was provided (Schwarz et al. 2013) (Fig. 4). Also in silico, the effect of external mechanical stimulation was negligible at 2 weeks (control: 14.75 mm^3^, only load: 14.69 mm^3^), 4 weeks (control: 27.75 mm^3^, only load: 27.76 mm^3^), and 6 weeks (control: 27.84 mm^3^, only load: 27.93 mm^3^) postoperation (Fig. 4).

### Immediate release of BMP-2 in a critical-sized bone defect results in predicted non-union

**Fig. 5:**
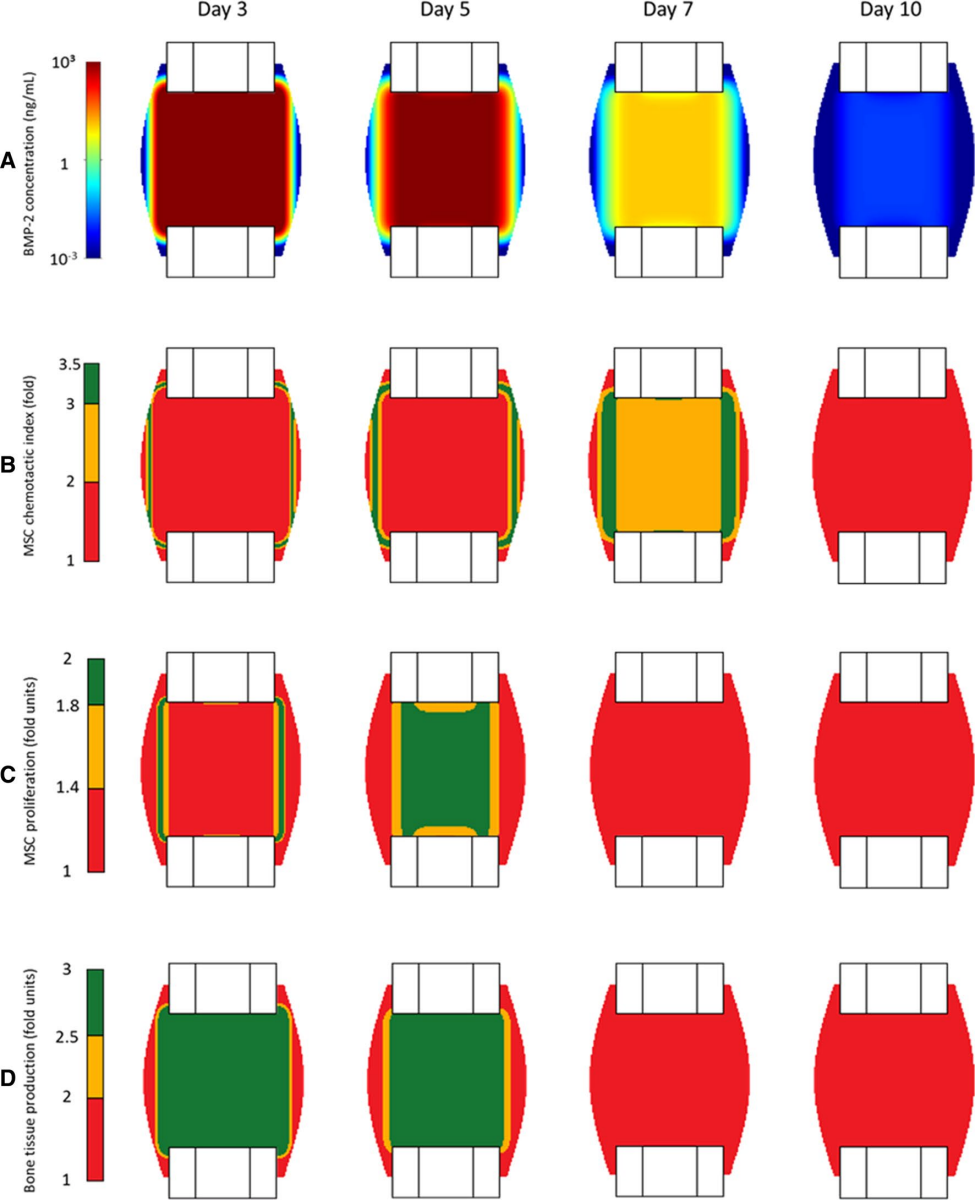
In silico predicted dynamics of BMP-2 concentration within the callus growth region (**A**) and its effects on the chemotactic attraction of MSCs (**B**), on enhancing MSC proliferation (**C**) and bone tissue production (**D**) at 3, 5, 7 and 10 days postoperation. The results refer to the simulation of a BMP-2 treatment instantly released. Note: The color scale is logarithmic for BMP-2 concentration plots

Computer model predictions of the fast release of BMP-2 (with no collagen sponge) showed a fast BMP-2 consumption, dropping to physiological levels in less than two weeks (Fig. 5A). On day 10 postoperation, the maximum concentration within the callus was predicted to be already below 1 ng/cm^3^ due to the fast degradation of the growth factor (Fig. 5A). The maximum effect of BMP-2 on cell chemotactic migration was limited to the first 7 days postoperation (in green in Fig. 5B). After the first week, the concentration of BMP-2 was predicted to rapidly decrease and its effect on chemotaxis was already over after 10 days (Fig. 5B). From day 7 on, BMP-2 had a negligible effect on the enhancement of MSC proliferation and bone tissue production within the healing region (Fig. 5C and D).

**Fig. 6:**
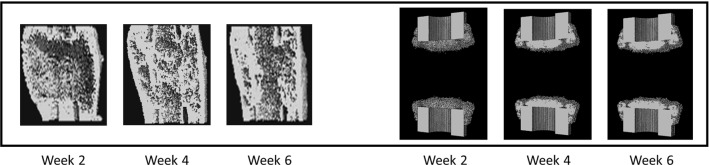
Bone tissue patterning of critical-sized defect healing under the only-BMP-2 condition (no external mechanical stimulation, exogenous BMP-2) at 2, 4, and 6 weeks postoperation when the gradual release of the growth factor is not implemented in the in silico model (right). Under this condition, the model is not able to reproduce BMP-2-treated defect healing as observed in the pre-clinical study (left) (Schwarz et al. 2013)

Under BMP-2 treatment, bone tissue patterning in µCT showed periosteal bridging already after 2 weeks (Fig. 6). In silico simulations of bone healing under BMP-2 instantaneous release resulted in a non-union after 6 weeks. In silico predictions of bone patterning resembled the one predicted when the defect was not treated with BMP-2 (Fig. 6).

### Enhanced chemotaxis can explain BMP-2 supported critical-sized bone defect healing

**Fig. 7:**
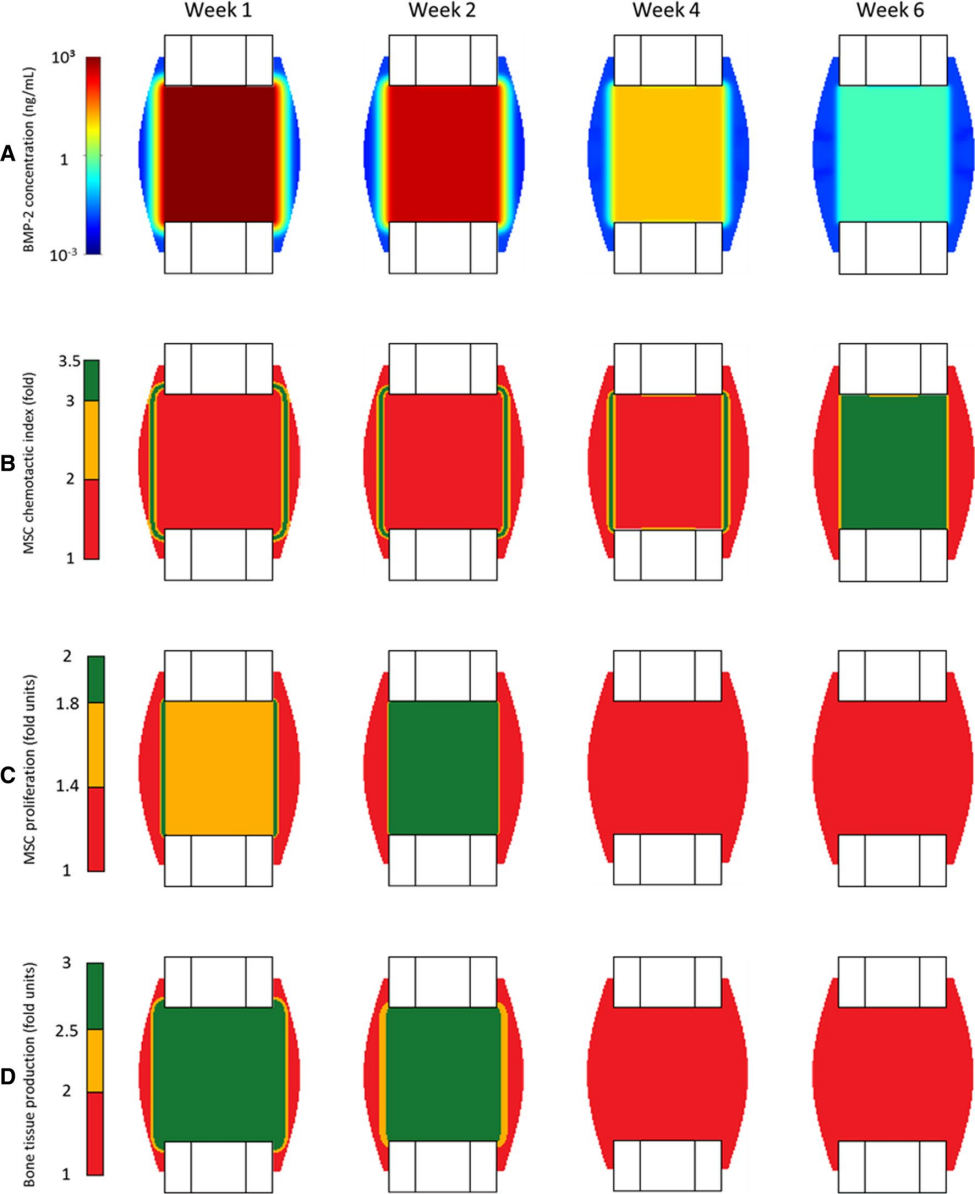
In silico predicted dynamics of BMP-2 concentration within the callus growth region (**A**) and its effects on the chemotactic attraction of MSCs (**B**), on enhancing MSC proliferation (**C**) and bone tissue production (**D**) at 1, 2, 4, and 6 weeks postoperation. The results refer to BMP-2 treatment when a collagen sponge where a gradual release was simulated. Note: The color scale is logarithmic for BMP-2 concentration plots

Computer model predictions of the release of BMP-2 from a collagen sponge inserted in the bone defect showed that the fast degradation of BMP-2 was compensated by the gradual release of BMP-2 to the callus growth region from the collagen sponge during the whole healing period (Fig. 7A). Until week 4 postoperation, the chemotactic effect of BMP-2 on MSC migration was predicted to have maximal impact in a region situated in the periosteal callus joining both bone ends (Fig. 7B). This region of maximal effect was predicted to slowly move from the periphery of the callus to the inside of the defect over time (Fig. 7B). Similarly, computer model predictions of BMP-2 release from the collagen sponge showed that BMP-2 had a maximum effect on enhancing MSC proliferation at the outer callus joining both bone ends during the first week postoperation. At 2 weeks postoperation, this region had shifted to the intercortical region (Fig. 7C). BMP-2 was predicted to stop promoting cellular proliferation from week 4 onward (Fig. 7C). BMP-2-enhanced bone tissue production within the defect gap in the first 2 weeks postoperation, when BMP-2 concentration was maximal. In the following weeks, BMP-2 concentration was predicted to reach physiological levels and reduce its influence on bone formation (Fig. 7D).

**Fig. 8:**
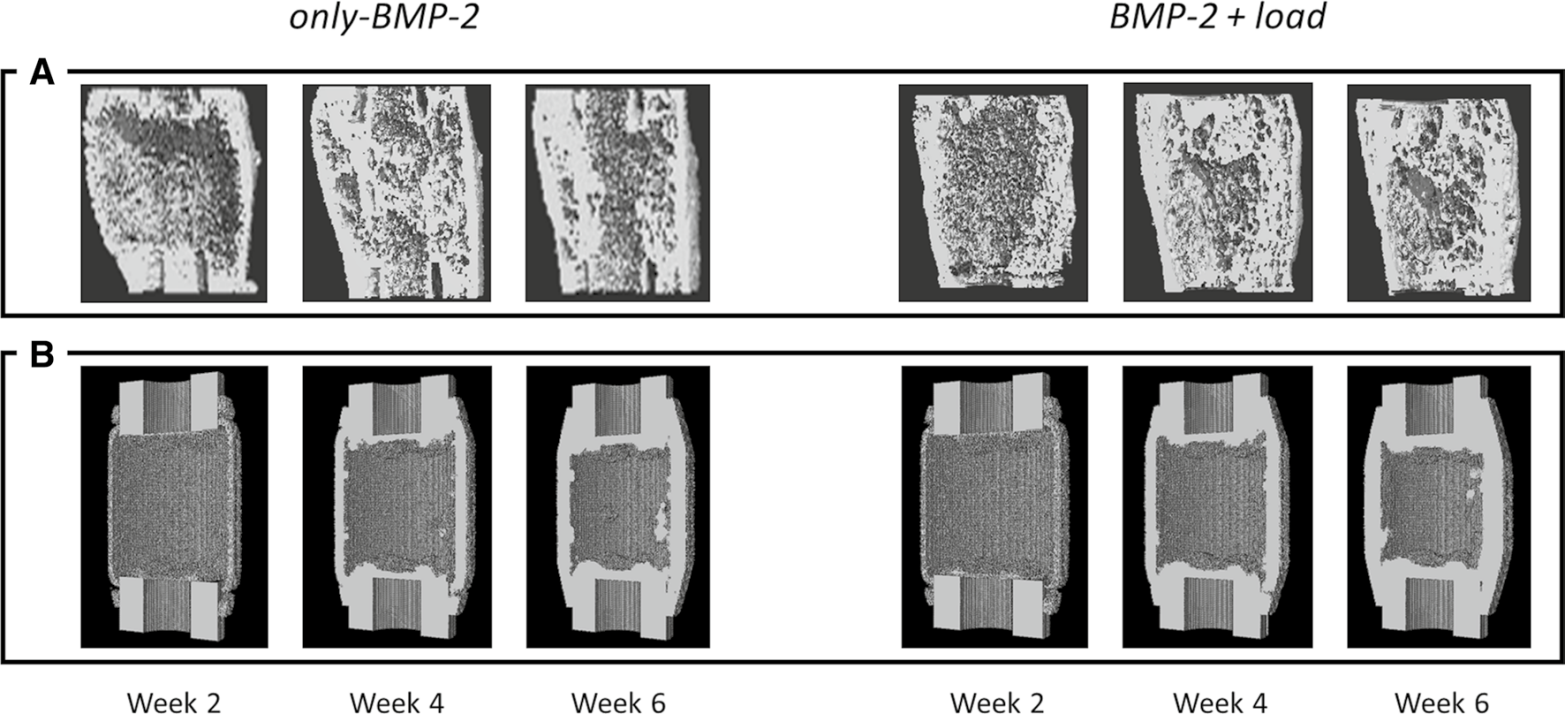
Bone tissue patterning of critical-sized defect healing under treated conditions (only-BMP-2 and BMP-2 + load cases) at 2, 4, and 6 weeks postoperation. Comparison between µCT images (Schwarz et al. 2013) (**A**) and in silico predictions (**B**)

Following experimental observations, computer model predictions of bone defect healing under gradual release of BMP-2 from a collagen sponge showed periosteal bony bridging already after 2 weeks (Fig. 8). In this model, a prolonged release of BMP-2 from the collagen sponge produced a chemotaxis gradient that drove the migration of progenitor cells toward the periosteal region where conditions were favorable for bone formation (Fig. 8B). This effect was in principle independent from an additional mechanical stimulation (Fig. 8A) (Schwarz et al. 2013).

**Fig. 9:**
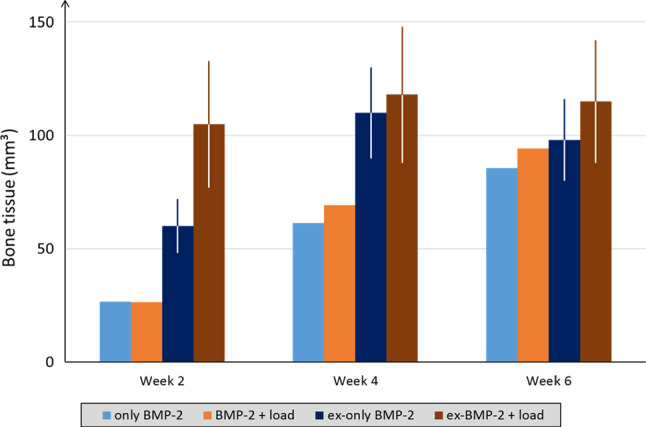
Mineralized callus volume comparison between in silico (average) and in vivo (BV average ± SD) within the healing region at 2, 4, and 6 weeks postoperation for BMP-2 treated case scenarios (only BMP-2 and BMP-2 + load). Note: “ex-” prefix in legend identifies in vivo

While in vivo external mechanical stimulation was able to slightly boost bone formation (Schwarz et al. 2013), in silico predictions did not show any additional effect of mechanical loading on bone formation (Fig. 8). Despite the additional loading generated higher intensity strains within the healing region (Supplementary data), its influence had a minor role at biological level. Quantitatively, predicted mineralized callus volume was similar between the BMP-2 treatment alone and the combination with mechanical stimulation after 2 (only BMP-2: 26.65 mm^3^, BMP-2 + load: 26.55 mm^3^), 4 (only BMP-2: 61.27 mm^3^, BMP-2 + load: 69.21 mm^3^), and 6 weeks (only BMP-2: 85.62 mm^3^, BMP-2 + load: 94.21 mm^3^) postoperation (Fig. 9). The model results disagree with the experiments, where significant BV differences were observed at 2 weeks postoperation (Fig. 9).

## Discussion

In this study, we investigated potential mechanisms behind bone tissue formation patterning during BMP-2-enhanced bone healing in a critical-sized bone defect using a mechano-biological computational model. Experimental studies have previously shown distinct tissue formation patterns under this treatment condition (Kokubo et al. 2004; Wulsten et al. 2011; Schwarz et al. 2013; Koolen et al. 2019). Moreover, it was shown that additional mechanical stimulation of critical-sized bone defects was able to further promote bone tissue formation under BMP-2-stimulated conditions in the first stages of healing (Schwarz et al. 2013). Since all these experimental observations were limited to the tissue level, we use a multiscale approach to investigate the effect of BMP-2 and mechanical signals on cellular behavior and its consequences at the tissue level. Computer model predictions showed a strong chemotactic effect of BMP-2 on MSCs toward the periosteal region, which was able to explain the periosteal bridging already observed after two weeks postoperation.

The proposed in silico approach combined the potentialities of FE analysis to investigate the continuous field of tissue mechanics and agent-based models to discretize the cellular and extracellular environment and has a detailed analysis of the spatiotemporal dynamics at those levels. The fast analysis provided by specialized FE software solved the differential equations that describe the mechanical behavior of the healing region. Both the natural gait of the animal and the additional exogenous loading stimulation were taking into account. To perform a fast analysis without losing accuracy within the region of interest, two different element sizes were used to mesh the bone structure and the callus growth region. For this latter, a finer mesh was used to increase the accuracy, while a wider mesh was used for the remaining model (cortical bone, marrow, external fixator). The mechanical environment was characterized by the temporal variation of stresses and fluid flow velocity within the callus growth region, which regulate the cellular behavior according to known mechanobiological rules (Checa et al. 2011). Those variations, simulated by the agent-based model, are reported in this model as differentiation of MSCs into other cell phenotypes. No other signals are considered to drive the differentiation of MSCs into other repair cells. Temporal changes in the mechanical environment within the healing region depend on the production of tissue matrix by the cells, which is simulated with the agent-based model.

The computer model showed its potentiality by reproducing the mechanical and biological environments of the pre-clinical study that already investigated bone defect healing under the stimulatory effect of BMP-2 (Schwarz et al. 2013). While bone healing was experimentally investigated at the tissue level, the computer model allowed to simulate the behavior at smaller scales (cellular and extra-cellular). Computer models are furthermore characterized by substantial flexibility that allows to investigate different experimental setups by simply adapting the geometries and algorithms to the desired case scenario. Multiple bone geometries, fixation strategies, or other therapeutics possibilities could be then investigated in the future by adapting the current model of bone defect healing.

### Control cases

The most remarkable observation between the case studies that did not involve BMP-2 treatment (control and only-load cases) was that the previously reported altered mechanical environment in critical-sized bone defects (Mehta et al. 2012) was not able to explain, alone, the non-union healing outcome observed in vivo (Schwarz et al. 2013). Even when our model predicted reduced mechanical strains in the critical-sized bone defect compared to a normal gap, the reduction in mechanical signals within the healing region was not able to predict alone the experimentally observed non-union. The resulting healing process was delayed, but the bone formation was not predicted to stop, generating the conditions for late healing. Indeed, if the computer model was run for longer than week 6 postoperation, successful healing would be achieved. Only when challenging biological conditions were implemented, such as a reduction of MSC recruitment, non-union was predicted. Compromised cellular recruitment could be representative of unsuccessful healing in critical-sized bone defects. This is highlighted in different studies where reduced recruitment of cells is identified as a possible healing deterrent of critical-sized defects (Bajada et al. 2009; Gómez-Barrena et al. 2020). The reduction in MSC recruitment was implemented in the model as a zeroing of MSC migration and proliferation after 10 days postoperation. The 10-day threshold was selected as a possible limit between the inflammatory and repair stage. As the former was not investigated, the critical-sized bone defect alterations of the inflammatory response are here represented as a stop of cell recruitment/proliferation from that time point onwards. A slightly earlier (time < 10 days) or later (time > 10 days) arrest of simulated cell recruitment will just result in the prediction of a slightly earlier or later inhibition of bone repair, but would not alter the conclusion that reduced mechanical signals within the healing region in critical-sized bone defects alone cannot explain the mechanobiological regulation of critical-sized bone defect healing, based on state-of-the-art knowledge. The use of nanoparticles (Wang et al. 2019) or specific chemokines (Liu et al. 2015) to promote cell migration to the healing region has been proposed as a therapeutic strategy. Limited recruitment of MSCs and the low mechanical signals within the healing region create the right environment for the encapsulation of the marrow cavity.

The computer model predicted negligible effects of additional weekly mechanical stimulation in untreated (no BMP-2) critical-sized bone defect healing. This agrees with in vivo observations, where significant differences in bone tissue volume between an untreated critical-sized bone defect and a weekly stimulated (mechanically) defect were not observed (Schwarz et al. 2013). The additional mechanical loading was provided, both in vivo and in silico, only once a week. Computer model predictions showed a minimal effect of this additional load on cell differentiation. The effect of mechanical stimulation on bone healing has been shown to depend on the frequency of tissue mechanical stimulation (Wolf et al. 2001; Judex et al. 2007) and the onset of loading during the healing process (Claes et al. 2009; Willie et al. 2011). Cyclic provision of the loading generates favorable conditions for healing when compared with static force (Lanyon and Rubin 1984; Carter 1987). Previous studies have shown successful critical-sized bone defect healing under daily mechanical stimulation (Zhao et al. 2009; Liu et al. 2018). The mechanical stimulation of the in silico model reproduced the weekly loading scenario applied in vivo (Schwarz et al. 2013). However, the dynamization effect observed to promote therapeutics in critical-sized defect had a minor role in this model. A mechanical stimulation signal closer to the one used in Schwarz et al. (2013) should be used in the future to get a better fit with experimental data.

### BMP-2 cases

Without the support of BMP-2 treatment, in silico bone healing predictions resulted in bone formation within the proximities of the defect extremities since the first iterations, leading to the encapsulation of the marrow cavity. This outcome agreed with experimental observations (Schwarz et al. 2013). The model predicted an unregulated migration of MSCs from the medullary cavity to the fracture gap and their immediate differentiation into osteoblasts, due to low interfragmentary strains. BMP-2 treatment instead led to computer model predictions of periosteal bony bridging already at 2 weeks postoperation, which agreed with experimental observations (Schwarz et al. 2013). Bone tissue was predicted to form periosteally from both cortical extremities of the defect. This periosteal bone formation occurred as a consequence of the non-monotonic relationship between BMP-2 concentration and cellular activities (Ribeiro et al. 2015). The maximal BMP-2 effect on MSC chemotaxis and proliferation was not located in regions with the highest BMP-2 concentration. The efficacy of BMP-2 on bone regeneration does not increase proportionally with its concentration; instead, the optimal dosage tends to be low (Schmidt-Bleek et al. 2016). For example, the BMP-2 chemotaxis effect was predicted to be the highest in the periosteal region during the early healing phases, where the predicted BMP-2 concentration was close to 1 ng/cm3 (Lind et al. 1996; Fiedler et al. 2002). As a result, MSCs were attracted to this region, leading to periosteal bone formation and healing through periosteal bridging (Fig. 8). The in silico results under BMP-2 treatments highlight chemotaxis as a key process toward successful healing in critical-sized bone defects. Alternative treatment strategies could include analogue chemotactic effects to support the periosteal bridging formation within reasonable healing time.

### Collagen sponge

Additional analyses were performed to investigate the importance of the BMP-2 gradual release by the collagen sponge. Computer model predictions showed that freely available BMP-2 within the defect quickly reaches physiological values due to its rapid degradation. After 1 week, under freely available BMP-2 conditions (no sponge), BMP-2 did not have any effect on cell migration and proliferation (Fig. 7), leading to a non-union. These results agree with experimental studies showing more effective defect healing when BMP-2 is delivered during a longer period (Visser et al. 2009; La et al. 2010; Pelaez et al. 2014).

### Limitations

The model was limited in the number of processes we considered to be stimulated by BMP-2 treatment. The effect of BMP-2 on bone healing was simulated considering the same factors previously investigated by Ribeiro et al. (2015). The advantage is that Ribeiro et al. already used those factors to investigate BMP-2 supported bone healing in a completely different animal model. They showed the power of computer modeling approaches to estimate the multiscaled effect of BMP-2 on the evolution of large bone defects healing. However, both here and in Ribeiro et al. chondrocytes or fibroblasts were not affected by BMP-2. Moreover, no enhancement of MSC differentiation or osteoblastogenesis was taken into account. Future models should extend the investigation to other biological aspects that could enhance bone healing under BMP-2 treatment, for example promotion of MSC differentiation toward osteoblasts (Reddi 1998), chondrocyte maturation (Shu et al. 2011). Besides, treatment methodology should be further investigated by exploring more sponge release dynamics and the provision of exogenous cells within the defect opening, in support to the therapeutic strategy. In addition, as the focus of this study was the tissue patterning evolution within the callus growth region, tissue formation inside the marrow niche was excluded from the simulation. This limitation was introduced to reduce the computational complexity of the model, but it could be connected with its limited capacity to reproduce the significant production of bone tissue observed in vivo in the BMP-2 + load case scenario. Additionally, this model does not include the role of angiogenesis in the overall healing process. Although it is known that revascularization has a role in the regulation of healing, to avoid further complexity the process itself was not taken into account. We assumed that if the conditions for bone tissue formation were favorable, blood vessels would be already present at those locations. We simulated cell recruitment arrest few days postoperation, which might partially represent limited angiogenesis in critical-sized bone defect healing (Gómez-Barrena et al. 2020). Future studies should additionally investigate how the revascularization is influenced by the altered mechanical environment of bone defects by including endothelial cells.

### Differences with experimental results

The current computer model was not able to explain some in vivo observations in critical-sized bone defects. For example, when compared with in vivo data (Schwarz et al. 2013), our model predicted more bone formation under untreated conditions and more bone resorption in the BMP-2 cases. This could be related to mechano-regulation aspects of the bone remodeling process that are not included in our model. Computer model predictions showed bone resorption in the endosteal and intercortical regions after a stiff periosteal external callus stabilized the fracture (BMP-2 cases). The inclusion of a “silent zone” in the mechano-regulation algorithm could better explain in vivo observations. Previous mechano-biological computer models of bone healing have already indicated a need to further investigate remodeling phase (Gerhard et al. 2009; Borgiani et al. 2019). While bone remodeling is not the focus of the current study, some discrepancies in the results can be explained from the lack of an algorithm to simulate the dynamic balance between bone formation and resorption. Bone remodeling is a process that can last from some months up to years until the original shape and functionality of the bone are fully restored (Ghiasi et al. 2017). The current model did not capture the remodeling phase of healing. Thus, the remodeling phase should be further investigated. Previous studies have implemented algorithms to investigate the mechanical regulation of bone remodeling during healing. Most of these models have implemented either a reduction of bone density under increasing presence of osteoclasts (Scheiner et al. 2013) or low mechanical strains (Weinkamer et al., 2019) or the removal of bone tissue in regions where mechanical stimuli were under a defined threshold (Borgiani et al. 2019). Recently, an in silico model was published to describe mechano-regulated biochemical effect on osteoblasts and osteoclasts remodeling activities (Kameo et al. 2020). Future studies should investigate the potential of these algorithms to explain bone remodeling during BMP-2 supported bone regeneration.

Besides, the BMP-2 treated bone defect model did not show the positive effect of external loading on bone tissue formation already after 2 weeks. Experimentally, BMP-2 treatment of bone defect healing was more effective when external weekly mechanical loading was additionally provided (Glatt et al. 2012; Schwarz et al. 2013). This promoted bone formation within the defect and resulted in faster stabilization of the defect (Schwarz et al. 2013). We simulated the same in vivo conditions (Schwarz et al. 2013) to investigate how mechanical stimulation further enhances BMP-2 treated bone healing. Computer model predictions showed no further bone tissue formation related to external loading at 2 weeks, but they showed an effect at later time-points. Besides, the amount of bone predicted under BMP-2-stimulated conditions was lower than experimentally observed, although the same tissue patterns were predicted. This could be explained by an effect of BMP-2 on osteogenesis, which was not included in the model.

Despite these limitations, the developed computer model was able to identify several potential mechanisms involved in critical-sized bone defect healing: (1) A potential limited biological activity in terms of cellular recruitment, which can explain non-unions in untreated critical-sized bone defects; (2) a potential early differentiation of periosteal and marrow-derived MSCs toward osteoblasts, due to low mechanical signals, which can explain bone marrow encapsulation since the initial phases of healing; (3) BMP-2-induced periosteal bony bridging, which can be explained by the chemotactic effect of BMP-2 on MSCs.

## Conclusions

In conclusion, using in silico analyses we were able to demonstrate that:

- Low mechanical signals within the healing region predicted by finite element analysis of critical-sized bone defects resulted in delayed healing. Simulated limited cellular recruitment could explain non-union in an untreated critical-sized bone defect;
- the computer model predicted healing of critical-sized defects under BMP-2 treatment when a collagen sponge-driven BMP-2 release was implemented in the model;
- BMP-2-stimulated chemotaxis played the main role in the regulation of cellular dynamics during critical-sized bone defect healing and the early promotion of periosteal bone bridging.

Computer model predictions identified BMP-2-driven chemotaxis as a key mechanism behind experimentally observed bone tissue formation. While BMP-2-driven increases in proliferation and bone formation did not have strong effects, enhanced migration of progenitors to this specific location allowed bone bridging in the periosteal region, resulting in defect healing within weeks. Furthermore, the computer model could explain the role of a slow-release BMP-2 carrier on the healing outcome. In the future, this model will be further developed to optimize treatment strategies and to further investigate mechanisms behind impaired regeneration.

## Supplementary Information

Below is the link to the electronic supplementary material.

Supplementary file1 (TIF 717 kb)

## Data Availability

The raw and analyzed data can be made available by the authors to any researcher.

## Code availability

The code can be made available by the authors to any researcher.
