# Supplementary figures and images for: Bone morphogenetic protein 2-induced cellular chemotaxis drives tissue patterning during critical-sized bone defect healing: an in silico study
Source: Biomech Model Mechanobiol. 2021 May 28;20(4):1627–44. doi: 10.1007/s10237-021-01466-0 (PMC8298257; doi:10.1007/s10237-021-01466-0)

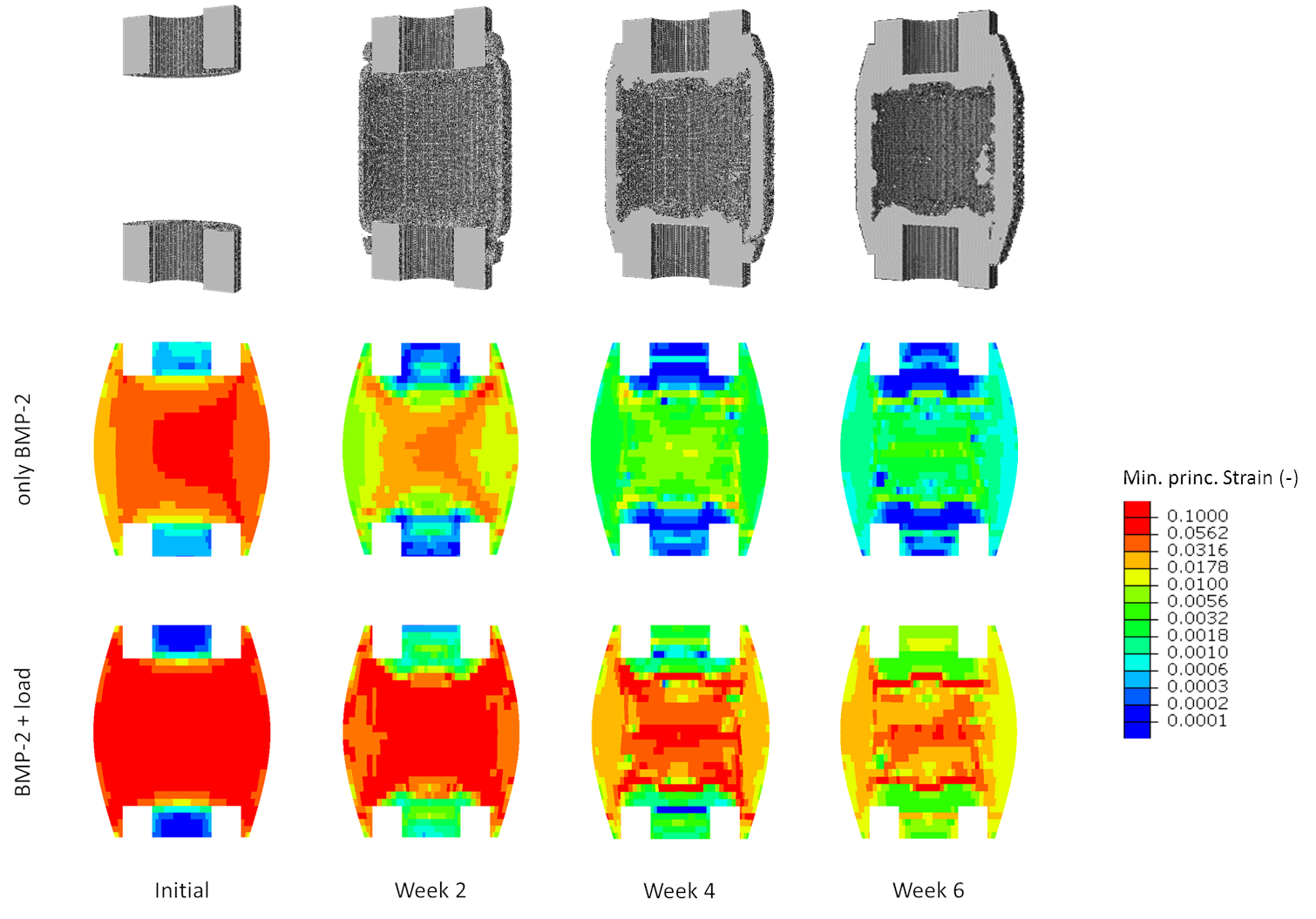

Supplement: Supplementary file 1 — Supplementary file1 (TIF 717 kb) [file 10237_2021_1466_MOESM1_ESM.tif]
